# Supplementary material for: A proline deletion in IFNAR1 impairs IFN-signaling and underlies increased resistance to tuberculosis in humans
Source: Nat Commun. 2018 Jan 8;9:85. doi: 10.1038/s41467-017-02611-z (PMC5758831; doi:10.1038/s41467-017-02611-z)
Supplement: Supplementary file 1 — Supplementary Information [file 41467_2017_2611_MOESM1_ESM.pdf]

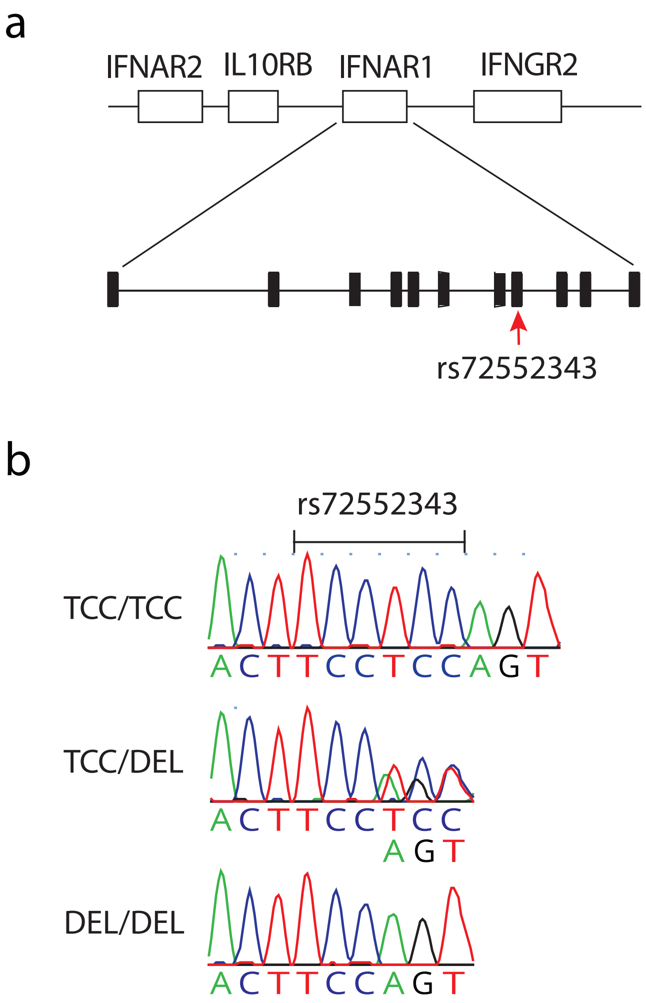

Supplementary Fig. 1. Schematic genome localization and sequence variations of IFNAR1 variant SNP rs72552343. (a) Genome organisation of chromosome 21q22.1 genes with the location of the identified SNP rs72552343. (b) Electropherograms of identified sequence variations of SNP rs72552343 demonstrating the deletion of TCC in IFNAR1. The data shown are representative of 3 individual donors / genotype.

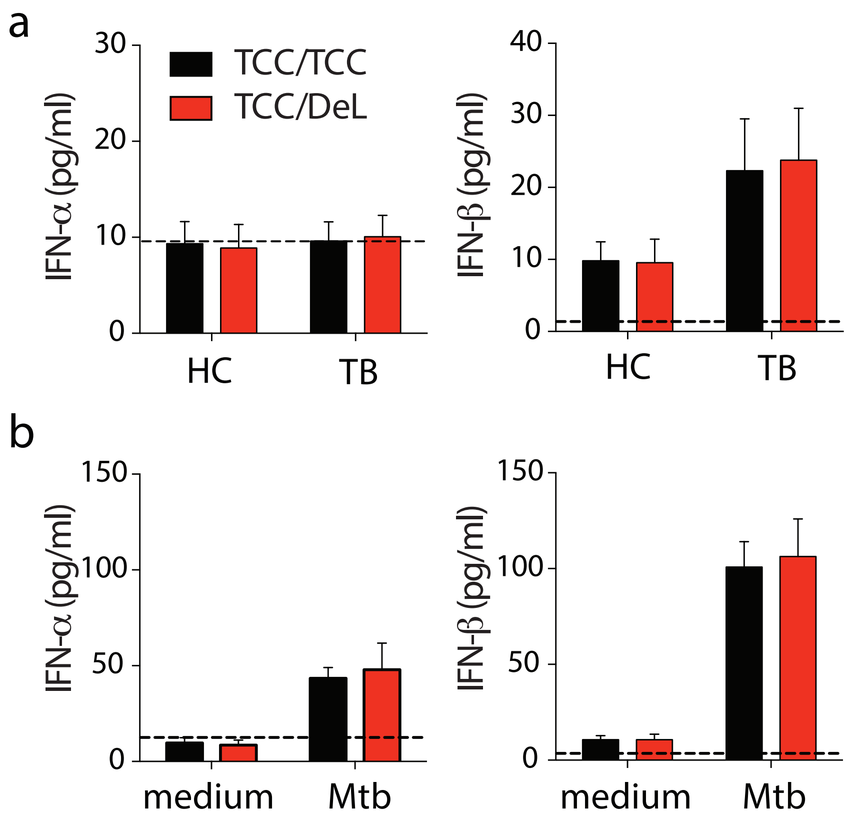

Supplementary Fig. 2. TCC deletion in IFNAR1 does not impair type I IFN production in TB patients or *M. tuberculosis*-infected macrophages. (a) IFN-α and IFN-β levels in the plasma of HC and TB patients carrying TCC/TCC or TCC/Del genotype determined by ELISA. Data shown are mean cytokine concentration ± SD (n = 12 individuals / group). (b) IFN-α and IFN-β levels in the supernatants of uninfected or 24-hour *M. tuberculosis*-infected macrophage cultures determined using ELISA. Macrophages were differentiated from circulating monocytes isolated from TCC/TCC or TCC/Del healthy donors. Data shown are mean cytokine concentration ± SD (n = 6 individuals / genotype). The dotted lines depict the limit of detection of the cytokine ELISAs. Black bars: TCC/TCC and red bars: TCC/Del.

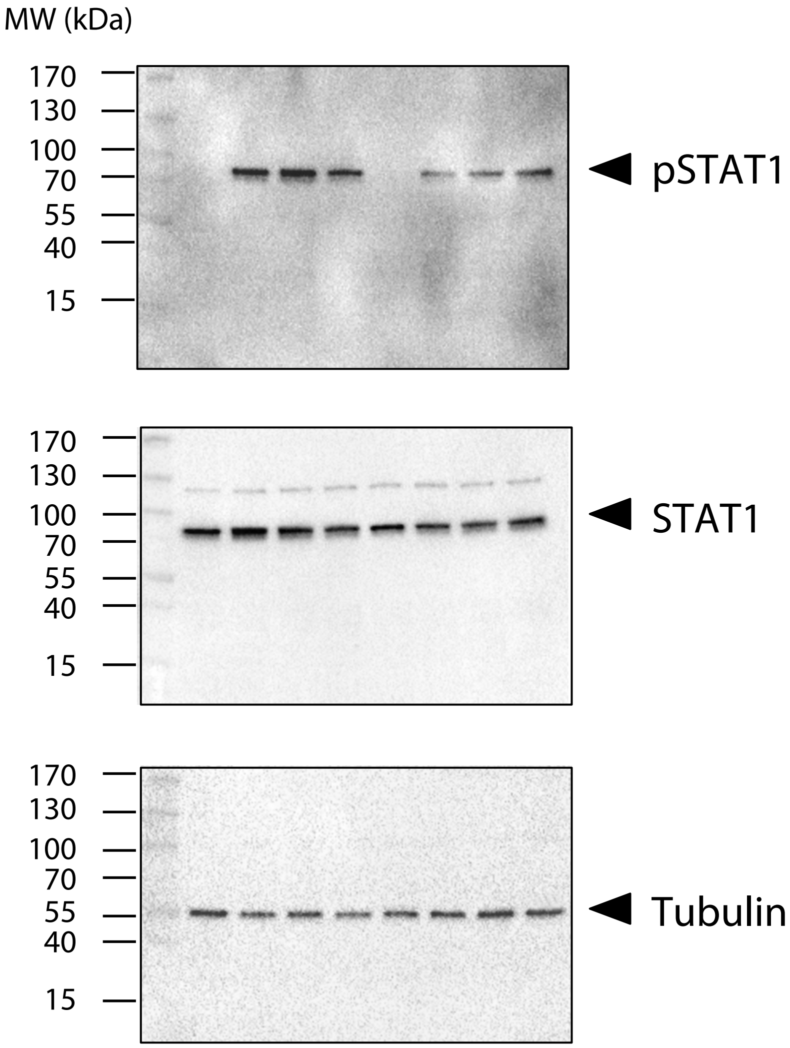

Supplementary Fig. 3. Full unedited gel images used for Fig. 4c. Arrow heads indicate the protein bands shown in Fig 4c.

Supplementary Table 1. Association of IFNAR1 SNPs and TB susceptibility (Discovery cohort)

			**Multiplicative**		**Additive**		**Dominant**		**Recessive**	
**SNP ID Genotype**	**HC** [*]	**TB**	***P*** [**]	**OR (95%CI)** [***]	***P***	**OR (95%CI)**	***P***	**OR (95%CI)**	***P***	**OR (95%CI)**
**rs2834191**										
TT	1408 (97.4)	1487 (97.0)	0.41	1.20 (0.78-1.85)	—	—	0.46	1.17 (0.75-1.82)	—	—
TG	37 (2.6)	45 (2.9)			—	—				
GG	0 (0.0)	1 (0.1)			Ref.	Ref.				
**rs1012334**										
AA	494 (34.2)	526 (34.3)	0.54	0.96 (0.87-1.07)	0.44	0.92 (0.74-1.13)	0.94	0.99 (0.85-1.15)	0.31	0.90 (0.75-1.09)
AT	677 (46.9)	738 (48.1)			0.30	0.90 (0.73-1.09)				
TT	274 (18.9)	269 (17.6)			Ref.	Ref.				
**rs17875752**										
GG	519 (35.9)	541 (35.3)	0.78	1.02 (0.91-1.12)	0.84	1.02 (0.81-1.27)	0.72	1.02 (0.88-1.19)	0.96	1.00 (0.82-1.23)
GT	712 (49.3)	764 (49.8)			0.94	0.99 (0.80-1.22)				
TT	214 (14.8)	228 (14.9)			Ref.	Ref.				
**rs72552343**										
TCC/TCC	1378 (95.4)	1499 (97.8)	**0.0002**	0.46 (0.31-0.70)	—	—	**0.0003**	0.47 (0.30-0.71)	—	—
TCC/DEL	66 (4.6)	34 (2.2)			—	—				

DEL/DEL	1 (0.0)	0 (0.0)			Ref.	Ref.				
**rs2843710**										
CC	626 (43.3)	691 (45.1)	0.42	0.95 (0.85-1.06)	0.64	0.94 (0.73-1.21)	0.33	0.93 (0.80-1.07)	0.85	0.97 (0.77-1.24)
CG	670 (46.4)	687 (44.8)			0.90	1.01 (0.79-1.30)				
GG	149 (10.3)	155 (10.1)			Ref.	Ref.				
**rs1041868**										
GG	521 (36.1)	560 (36.5)	0.47	0.96 (0.86-1.06)	0.37	0.90 (0.73-1.12)	0.78	0.97 (0.84-1.13)	0.31	0.90 (0.74-1.10)
GA	682 (47.2)	737 (48.1)			0.33	0.90 (0.73-1.11)				
AA	242 (16.7)	236 (15.4)			Ref.	Ref.				

*Number of samples with genotype frequency shown in parentheses.

**Significant *p* values (< *0.05*) are shown in bold.

***OR = odds ratio, numbers in parentheses following OR are 95% confidence intervals.

Supplementary Table 2. Characteristics of study populations

Cohort	Group	Number	Age (mean ± SD)	Sex (Male / Female)
**TB (discovery)**	HC	1445	36.84 ± 19.71	882/563
	TB	1533	36.92 ± 14.09	980/553
**TB (validation)**	HC	1084	40.54 ± 10.69	629/455
	TB	832	41.10 ± 19.04	496/336
**HepB**	HC	894	38.63 ± 14.91	502/392
	HepB	848	38.96 ± 14.30	557/291

Supplementary Table 3. Association of IFNAR1 rs72552343 SNP and pulmonary and extra-pulmonary TB

					PTB vs. HC		ETB vs. HC		ETB vs. PTB	
**Cohort**	**Genotype**	**HC** *	**PTB**	**ETB**	***P*** **	**OR (95%CI)** ***	***P***	**OR (95%CI)**	***P***	**OR (95%CI)**
**Combined**	TCC/TCC	2421 (95.7)	1762 (97.9)	556 (98.2)	**<0.0001**	0.47 (0.32-0.68)	**0.005**	0.40 (0.21-0.77)	0.66	0.85 (0.42-1.73)
	TCC/DEL	107 (4.2)	37 (2.1)	10 (1.8)						
	DEL/DEL	1 (0.1)	0 (0.0)	0 (0.0)						

*Number of samples with genotype frequency shown in parentheses

**Significant *p* values (< *0.05*) are shown in bold (dominant model).

****OR* = odds ratio, numbers in parentheses following OR are 95% confidence intervals.

Supplementary Table 4. Association of IFNAR1 rs72552343 SNP and pulmonary TB susceptibility stratified by mycobacterial culture positivity

					**Culture- vs. HC**		**Culture+ vs. HC**		**Culture- vs. Culture+**	
**Cohort**	**Genotype**	**HC***	**PTB (Culture-)**	**PTB (Culture+)**	***P*** **	**OR (95%CI)** ***	***P***	**OR (95%CI)**	***p***	**OR (95%CI)**
**Combined**	TCC/TCC	2421 (95.7)	741 (98.0)	1021 (97.9)	**0.004**	0.45 (0.26-0.78)	**0.002**	0.48 (0.30-0.76)	0.85	0.93 (0.48-1.82)
	TCC/DEL	107 (4.2)	15 (2.0)	22 (2.1)						
	DEL/DEL	1 (0.1)	0 (0.0)	0 (0.0)						

*Number of samples with genotype frequency shown in parentheses

**Significant *p* values (< *0.05*) are shown in bold (dominant model).

****OR* = odds ratio, numbers in parentheses following OR are 95% confidence intervals.

Supplementary Table 5. Association between IFNAR1 rs72552343 SNP and pulmonary TB susceptibility stratified by sputum smear AFB positivity

					AFB- vs. HC		AFB+ vs. HC		AFB- vs. AFB+	
**Cohort**	**Genotype**	**HC** *	**PTB (AFB-)**	**PTB (AFB+)**	***P*** **	**OR (95%CI)** ***	***P***	**OR (95%CI)**	***P***	**OR (95%CI)**
**Combined**	TCC/TCC	2421 (95.7)	1211 (98.0)	551 (97.9)	**0.0005**	0.46 (0.29-0.71)	**0.02**	0.48 (0.26-0.89)	0.88	0.94 (0.47-1.90)
	TCC/DEL	107 (4.2)	25 (2.0)	12 (2.1)						
	DEL/DEL	1 (0.1)	0 (0.0)	0 (0.0)						

*Number of samples with genotype frequency shown in parentheses

**Significant *p* values (< *0.05*) are shown in bold (dominant model).

****OR* = odds ratio, numbers in parentheses following OR are 95% confidence intervals.

Supplementary Table 6. Association of IFNAR1 rs72552343 SNP and pulmonary TB susceptibility stratified by the presence or absence of pulmonary cavities

				Multiplicative		Dominant	
**Cohort**	**Genotype**	**Cavity-***	**Cavity+**	***P* ****	**OR (95%CI) *****	***P***	**OR (95%CI)**
**Combined**	TCC/TCC	1000 (97.3)	762 (98.8)	**0.02**	0.43 (0.20-0.90)	**0.02**	0.42 (0.19-0.90)
	TCC/DEL	28 (2.7)	9 (1.2)				
	DEL/DEL	0 (0.0)	0 (0.0)				

* Number of samples with genotype frequency shown in parentheses.

** Significant *p* values (< *0.05*) are shown in bold.

*** *OR* = odds ratio, numbers in parentheses following OR are 95% confidence intervals.

Supplementary Table 7. qRT-PCR primer sequences

Gene	Forward (5′-3′)	Reverse (5′-3′)
hIFNAR1	ATTTACACCATTTCGCAAAGCTC	TCCAAAGCCCACATAACACTATC
hSTAT1	CGGCTGAATTTCGGCACCT	CAGTAACGATGAGAGGACCCT
hSTAT2	CCAGCTTTACTCGCACAGC	AGCCTTGGAATCATCACTCCC
hCXCL10	GTGGCATTCAAGGAGTACCTC	TGATGGCCTTCGATTCTGGATT
hIRF1	CTGTGCGAGTGTACCGGATG	ATCCCCACATGACTTCCTCTT
hIFIT1	GCGCTGGGTATGCGATCTC	CAGCCTGCCTTAGGGGAAG
hMX1	GTTTCCGAAGTGGACATCGCA	CTGCACAGGTTGTTCTCAGC
hGAPDH	GCACCGTCAAGGCTGAGAAC	TGGTGAAGACGCCAGTGGA
hISG15	CGCAGATCACCCAGAAGATCG	TTCGTCGCATTTGTCCACCA
hOAS1	TGTCCAAGGTGGTAAAGGGTG	CCGGCGATTTAACTGATCCTG
mIrf1	ATGCCAATCACTCGAATGCG	CCTGCTTTGTATCGGCCTGT
mIsg15	GGTGTCCGTGACTAACTCCAT	CTGTACCACTAGCATCACTGTG
mOas1	GGGCCTCTAAAGGGGTCAAG	TCAAACTTCACTCCACAACGTC
m18S	GTAACCCGTTGAACCCCATT	CCATCCAATCGGTAGTAGCG
